# 

**Published:** 1869-10

**Authors:** 


### NEW BOOKS.

POLLARD'S LIFE OF DAVIS has been received. We have read it with much interest, and consider it by far the most entertaining book that has grown out of the war.

PETERSON.—The best and cheapest ladies' magazine published. Splendid fashion plates —useful patterns—household receipts and entertaining stories, by our most celebrated authors. Subscribe for it—only $2.00 per year. Address Charles J. Peterson. 306 Chestnut St., Philadelphia.

ZELL'S ENCYCLOPEDIA.—This work excludes the necessity for a Dictionary of Language, a Gazetteer of the World, a Biographical Dictionary of Nations, a Dictionary of Law and Medicine, for it is each and every one of them combined, in one splendid work, easy of reference, beautifully printed and illustrated, and should be in every man's library. It is having a wonderful sale, and agents are reaping large profits by it. Address T. Ellwood Zell, Philadelphia, for an agency.

PETERS' MUSICAL MONTHLY.—Should be in the hands of every musician. It contains more music for three dollars a year than can be purchased for ten times that sum elsewhere. It is a beautifully printed magazine that should find a lodgment on every lady's piano. Address J. L. Peters, 198 Broadway.

HARPER.—Those three delightful periodicals' Harper's Weekly, Bazaar and Monthly, come to our table regularly. We know of no way of investing ten dollars more profitably than to subscribe for all three of those useful journals. Take the Bazaar for your wife, the Weekly for your children, and the Magazine will furnish you any amount of valuable and profitable reading for the month. Address Harper Bros., N. Y.

STEELE'S SCHOOL BOOKS.—"Sixteen Weeks in Philosophy," "Sixteen Weeks in Chemistry" and "Sixteen Weeks in Astronomy," by Prof. J. D. Steele, of this city, have been laid upon our editorial table. We have not had sufficient leisure to examine these works as thoroughly as we would like, yet have satisfied ourselves that they are by far the best school books, upon the sciences named, now in use. All the recent discoveries in science are given, and the new phraseology adopted. The books are handsomely illustrated and beautifully printed. They are the most cheerful looking school books that we have ever seen. We are glad to note that they are being extensively adopted in the schools throughout the land.

MARK TWAIN'S BOOK.—We have received from the publishers, a copy of "The Innocents Abroad or the New Pilgrim's Progress," by Mark Twain.

We are sorry it reached us too late for an extended notice in this number of the BISTOURY. We are ready for the press and have only time to say that it is a beautifully printed and illustrated work of 650 pages—is the story of a pleasure excursion to the holy land—with descriptions of countries and natives and the adventures of the author, told in his humorous style. Mark Twain is our idea of a humorist. He is capable of amusing and instructing at one and the same time; his humor does not require the aid of bad spelling to render it effective—but the quaint manner in which he tells his story and the ludicrous positions in which he places men and things will cause one to laugh in spite of himself. A rich treat is in store for all who buy the book.

Sold by subscription only, and Agents are wanted in every County in the United States. Apply to, or write the "American Publishing Co.," 149 Asylum St., Hartford Conn., for an agency.

### Answers to Correspondents.

Under this head we will answer all communications addressed to us of interest to the general reader, and will reply to such persons who forget to enclose us a postage stamp.

We will cheerfully give our advice in this department to all who seek it, and solicit correspondence upon all subjects of interest to the people generally.

Mrs. W.—*West Bloomfield, N. Y.*—Call on Dr. Wood, of your place. He can tell you what the difficulty is.

Dr. R.—*Milo, N, Y.*--You will find an answer to your query in "Medical Items and News."

J.--*Auburn, N. Y.*—They are miserable quacks. The less you take of their medicine, the better. See our article on Quackery in this number.

J. R. S.—*Watertown, N. Y.*—You have cataract. It can be removed by means of a surgical operation. Medicine can do no possible good.

Dr. A.—*Syracuse, N. Y,*—Write Dr. L. A. Babcock, Freeport, Ill. He will give you the desired information. His uterine supporter is the best in use.

C——.—*Rome, N. Y.*--If, as you suppose, it is scirrhus of the breast, have it removed at once, surgically, before the glands in the axilla become tender.

P. R. L.--*Hartford, Conn.*—The Jefferson Medical College, in Philadelphia, we consider the best in this country. Tuition as cheap as in any first-class College.

P. W.—*Abbeville, Ala.*--On a par with every other scoundrel--a quack of the most villainous sort. Have nothing to do with him.

Mrs. J.--*Marion, S. C.*--This is the most favorable time of year to undergo a surgical operation. We can provide for you. Come at once.

J. R. & Co.--*Covington, Va.*--We don't deal in spectacles. Send your order to Collingwood & Strang, of this city.

P. W.—*Williamsport, Pa.*--Your child is not too young to have its foot straightened. Have it attended to at once, as it can now be done by an apparatus, and without cutting.

J. D.--*Fort Hays, Kansas.*—You forgot to inclose the necessary stamp to prepay postage. We therefore answer you here: Cannot tell whether your case is curable or not, without seeing you.

Mrs. J.--*Springfield, Ill.*—The opacity is caused by the "sugar of lead," wash you used in baby's eyes. It can never be removed. Never use such washes about the eyes again.

T. W.—*Yancton, Dacotah.*—You have a granular lid. The disease exists upon the under surface of the eye-lid. Cease plucking out the eye lashes--it will do no good. Apply to some Oculist for treatment.

Rev. J. H. B.--*Hillsboro, Texas.*--Cannot tell why gravel should be more prevalent with you than elsewhere. Are not sufficiently acquainted with your country to enable us to assign a reason.

Dr. R.--*South Bend, Ind.*--The *Medical Record*, published by Wm. Wood & Co., price $4 per year, or the Philadelphia *Med. and Surg. Reporter*, S. W. Butler, M. D., publisher, will meet your requirements.

J. R. D.--*Plattsburgh. N. Y.*--Your family physician is the proper person to apply to. He can probably tell whether it is an ovarian tumor or not. We can visit you if he thinks best.

Mrs. L.--*Manchester, N. H.*--The *caution* contained in the advertisement of the pills referred to, is intended to sell them. Have nothing to do with them. They will ruin your health. We have no remedy for your trouble, and wouldn't sell it if we had.

Dr. N.—*Meadville, Pa.*—First. "Stellwag on the eye," to be had of Wm. Wood & Co., N. Y. Second. No work of any value published on Catarrh. Third. We don't purpose publishing instrument makers gratuitously any longer.

Mrs. R.—*Blossburgh, Pa.*—If you will vouch for the truthfulness of what you say, we will publish the fact, with the physician's name, in the BISTOURY. Every miserable, contemptible abortionist in the land should be exposed. Help us do it.

Miss ——,--*Dunkirk. N. Y.*--If you have read the BISTOURY, you must have had our opinion of the crime you seek us to do. We will not do it ourself, and will certainly expose you if you have it done. You have no right to commit murder.

L.--*Cattskill, N. Y.*--Amaurosis is a very convenient term made use of by physicians. So very many different forms of blindness come under this head, that it is impossible for us to say whether your case is curable or not. Better call for an examination.

Mrs. S.--*Sandusky, O.*--If you are too poor to consult an Aurist, you had better syringe the child's ear, very carefully, twice a day, with soap-suds. Dissolve three grains of tannin in half an ounce of pure glycerine. Place two or three drops in the child's ear, every night confining it there with cotton wool. Discharges from the ear, caused by scarlet fever, are troublesome cases Be careful, or your child will be deaf.

